# AI-Driven Sensing Technology: Review

**DOI:** 10.3390/s24102958

**Published:** 2024-05-07

**Authors:** Long Chen, Chenbin Xia, Zhehui Zhao, Haoran Fu, Yunmin Chen

**Affiliations:** Department of Civil Engineering, Zhejiang University, Hangzhou 310058, China; 3210100898@zju.edu.cn (L.C.); 3210100343@zju.edu.cn (C.X.); zhaozh@zju.edu.cn (Z.Z.);

**Keywords:** sensing technology, ML/DL algorithm, performance enhancement, AI-driven sensing applications

## Abstract

Machine learning and deep learning technologies are rapidly advancing the capabilities of sensing technologies, bringing about significant improvements in accuracy, sensitivity, and adaptability. These advancements are making a notable impact across a broad spectrum of fields, including industrial automation, robotics, biomedical engineering, and civil infrastructure monitoring. The core of this transformative shift lies in the integration of artificial intelligence (AI) with sensor technology, focusing on the development of efficient algorithms that drive both device performance enhancements and novel applications in various biomedical and engineering fields. This review delves into the fusion of ML/DL algorithms with sensor technologies, shedding light on their profound impact on sensor design, calibration and compensation, object recognition, and behavior prediction. Through a series of exemplary applications, the review showcases the potential of AI algorithms to significantly upgrade sensor functionalities and widen their application range. Moreover, it addresses the challenges encountered in exploiting these technologies for sensing applications and offers insights into future trends and potential advancements.

## 1. Introduction

In the current era marked by swift technological evolution, sensing technology occupies a pivotal position in diverse sectors, including advanced industrial processes [1], robotics [2], biomedical engineering [3,4,5,6], and civil engineering [7,8]. These sensors employ sophisticated structural design [9,10,11,12] and innovative material optimization [13,14] in their sensitive units to transform stimuli from objects into electrical or optical signals. This conversion process is further refined through stages like signal amplification, filtering, and impedance matching, enhancing the signal’s quality, stability, and interoperability. However, despite continuous technological innovations, improvements in sensor accuracy, sensitivity, and adaptability still face bottlenecks due to the precision limitations of micro-nano fabrication processes [15], the pace of new material development and application [16], intrinsic noise limitations of circuit components [17], and the complexity and real-time requirements of signal processing algorithms [18].

These bottlenecks lead to a variety of unique and complex challenges across different application domains. For instance, in industrial automation, the precision and sensitivity of sensors on the production line affect the speed and accuracy of product line operations and defect detection, making sensors crucial for ensuring production efficiency and product quality [19]. In the realm of robotics, sensors are required to offer high accuracy while also possessing multifunctional adaptability, enabling robots, i.e., unmanned aerial vehicles [20] and deep-sea robots [21], to adapt to fluctuating work environments and tasks [22]. Similarly, in biomedical engineering and structural health monitoring, sensors are tasked with identifying subtle physiological or structural changes, ensuring both high precision and reliability even under complex or extreme conditions [23], such as monitoring physiological data of human skin during motion [24] and monitoring railway responses in permafrost regions [25].

In this context, the advent of machine learning and deep learning technologies stands as a crucial breakthrough in overcoming traditional technological constraints [26]. These cutting-edge algorithms uncover intricate patterns and correlations by autonomously analyzing vast data sets, thus optimizing sensor performance. They enhance sensor accuracy [27] and sensitivity [28] under specific conditions and bolster adaptability [29] to environmental shifts. More critically, beyond monitoring, these technologies enable efficient identification and predictive capabilities, heralding a new era in machine maintenance [30,31], disease diagnosis [32,33,34], structural damage prevention [35], and the environmental awareness and adaptability of robots [36,37,38]. Extensive research now concentrates on merging artificial intelligence with sensor technology [39], ranging from performance enhancement algorithms [40] and algorithm-driven device design [41] to broad applications in biomedical [42] and engineering fields [43].

Machine learning and deep learning’s contributions to sensing technology are segmented into four principal areas: sensor design, calibration and compensation, object recognition and classification, and behavior prediction. In this paper, we delve into the vital functions of artificial intelligence algorithms within these realms, highlighting the latest progress in innovative applications. This paper first discusses in Section 2 the role of artificial intelligence algorithms in guiding the sensor design. Subsequent sections, from Section 3, Section 4 and Section 5, explore the impact of algorithms on sensor calibration and compensation, object recognition and classification, and behavior prediction. The paper concludes by discussing the challenges of advancing sensing technology with these approaches and offers a forward-looking perspective on future trends.

## 2. Sensor Design Assisted by ML/DL

ML/DL assists in sensor design through two primary aspects. First, reverse engineering models, such as Artificial Neural Networks, are developed to design target sensor geometric configurations based on desired performance outcomes. Second, sensor performance is optimized during the design process through the use of algorithms like convolutional neural networks (CNNs), addressing issues such as small measurement ranges, low signal-to-noise ratios, and inadequate precision.

### 2.1. Inverse Design

Utilizing ML/DL algorithms for inverse design aims to economize fabrication costs by preventing excess sensor performance while simultaneously addressing the contradictory metrics of range and sensitivity, which are pivotal for sensor functionality. In this context, a refined method has been established for modeling capacitive pressure sensors using a functional link artificial neural network (FLANN). By employing FLANN, the approach precisely estimates the unknown coefficients in a power series expansion, capturing the sensor’s nonlinear response throughout its operational range. This estimation articulates a clear relationship between pressure input and sensor capacitance output, guiding the precise engineering of sensor parameters to achieve the intended performance profile [44]. Furthermore, the design of capacitive pressure sensors featuring micro-pyramidal electrodes and dielectrics demonstrates innovative customization for specific applications. The corresponding numerical model merges mechanical and electrodynamic analyses to predict the sensor’s pressure response across a wide dynamic range, enabling precise customization through an in-depth assessment of the sensor’s pressure range, linearity, and sensitivity. The incorporation of neural networks further enriches this design process by enabling a deep understanding of the interrelations between microstructural deformations and sensor performance, thereby guiding the creation of sensors with finely tuned responses to pressure variations [45].

**Figure 1:**
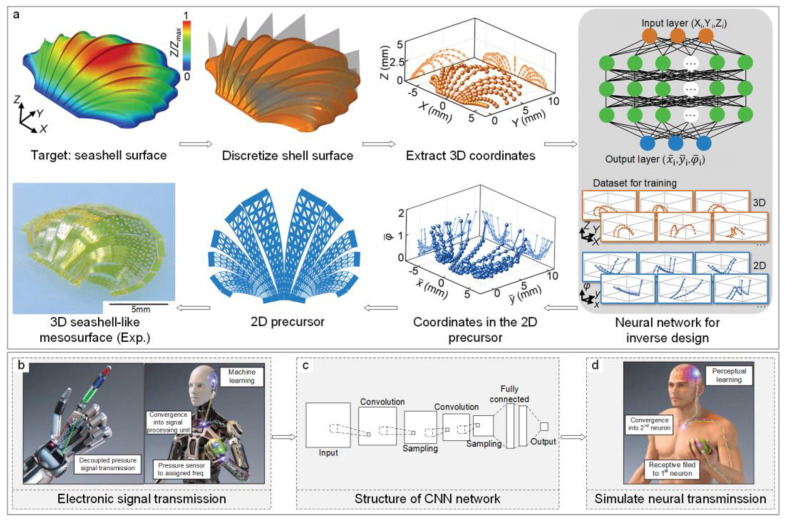
Application of ML/DL algorithms in sensor design. (**a**) Schematic illustrations of the inverse design process for a seashell mesosurface utilized in sensor integration. Adapted with permission. Copyright 2023, American Association for the Advancement of Science [41]. (**b**–**d**) Enhancing sensor signal processing through DL algorithm integration to simulate human neural transmission. (**b**) Left: depiction of a wireless parallel pressure cognition platform (WiPPCoP) on a robotic hand, capturing tactile signals simultaneously at unique frequencies. Right: implementation of WiPPCoP in a robotic system. Reproduced with permission. Copyright 2020, Wiley-VCH GmbH, Weinheim, Germany [37]. (**c**) Structure of a CNN trained with testing data. (**d**) Illustration of the human somatosensory system’s process for transmitting pressure sensations. Reproduced with permission. Copyright 2020, Wiley-VCH GmbH, Weinheim, Germany [37].

Simultaneously, the broad potential of ML/DL algorithms in inverse design notably extends to improving device adaptability across various environmental conditions. Xu Cheng employed biomimetic micro-lattice design strategies and inverse methods to assemble 2D films into targeted 3D configurations with diverse geometric shapes. By discretizing 3D surfaces and then leveraging a point-cloud-based CNN to map the point cloud data of complex 3D surfaces to 2D micro-lattice films, this approach predicted the point coordinates and corresponding porosity of 2D micro-lattice films, thus achieving the inverse design of complex 3D surfaces for specific applications [Figure 1a], such as a hemispherical electronic device optimized for cardiac sensing. This device, characterized by its adaptive geometry and optimized structural integrity, showcases the ability of ML-driven design to produce sensors that not only conform to dynamic operational contexts but also deliver precise measurements under varied conditions [41].

### 2.2. Performance Enhancement

Integrating machine learning algorithms into the signal processing phase of sensors can significantly enhance the accuracy of the devices. Samuel Rosset et al. used machine learning to detect pressure and its location on sensors, applying varied frequency signals to collect impedance and capacitance data. These data were analyzed to identify key statistical features, which were then processed using algorithms like K-nearest neighbors (KNNs), linear discriminant analysis (LDA), and decision trees (DTs). Their method achieved over 99% accuracy on a three-zone sensor for both location and pressure intensity [46]. Additionally, WiPPCoP, a novel wireless parallel signal processing technique, was developed for tactile signal management in robotics and prosthetic applications. This method began by collecting a vast amount of pressure signal data through wireless pressure sensors, which could be mounted on robot hands or other devices requiring pressure sensing [Figure 1b]. Based on pre-processed data, a CNN model was constructed to automatically learn the feature representation of pressure signals, facilitating classification or regression predictions of the signals [Figure 1c]. Regression predictions were used to forecast the continuous output of pressure signals. When trained with 100 data points, the CNN model demonstrated a mean squared error (MSE) and an error index of 0.12 and 0.09, respectively, indicating its applicability to real-world pressure signal processing tasks [Figure 1d]. In practice, the model could eliminate complex external wiring and monitor pressure at different locations in real-time. For instance, a trained CNN model could monitor pressure levels on a robot’s hand, aiding the robot in better task execution [37]. Further, Mehdi Ghommem et al. explored a microelectromechanical system (MEMS) sensor for detecting pressure and temperature, utilizing electrodes under a microbeam with direct and alternating voltage applications. Their design considered ambient temperature effects on the microbeam and air pressure impact on squeeze-film damping. A neural network trained on input data—comprising the first three natural frequencies of an arch beam at various temperatures, quality factors, and static deflection—enabled the detection of intertwined temperature and pressure outputs. Optimal temperature and pressure predictions, with RMSE values of 0.158 and 0.997, respectively, were achieved using leaky ReLU as the activation function [47].

Furthermore, ML/DL algorithms can enhance the limit of detection (LOD) for sensors. Experiments taking hydrogen concentration sensors as an example were conducted in six different metal channels (Au, Cu, Mo, Ni, Pt, Pd) for H_2_ sensing. By employing Deep Neural Networks (DNNs) and Gated Recurrent Units (GRUs) to train on the real-time noise signals of chemical sensors, a hidden relationship between hydrogen concentration and signal noise was established. This significantly improves the accuracy of gas sensors in detecting low concentrations of hydrogen [48].

Beyond electronic signal sensors, ML/DL algorithms are widely applied in fiber Bragg grating sensors for improving key parameters such as range, signal-to-noise ratio, and accuracy. When external pressure affects these sensors, the phase birefringence in the optical path changes, causing wavelength shifts in the interference spectrum. These shifts, encapsulating pressure variations, are characterized by tracking wavelength changes against pressure. A long short-term memory (LSTM) neural network model has been applied to convert recorded raw spectra into one- or two-dimensional data, enabling accurate pressure prediction. Experiments demonstrate the LSTM model’s superior accuracy over traditional machine learning methods, with a root-mean-square error (RMSE) of only 4.4 kPa within a 0–5 MPa range, thus allowing for precise fiber optic sensor measurements [49]. Similarly, a high spatial resolution flexible optical pressure sensor has been designed, where surface pressure affects the absorption and transmittance of reflected light between shielding and sensing layers, altering RGB values in corresponding images. Convolutional neural network (CNN) algorithms extract features from images to determine the force’s magnitude and location applied to the sensor, achieving an RMSE of about 0.1 mm for positioning and 0.67 N for normal force [50].

Fiber optic sensors, sensitive to both strain and temperature, face challenges with cross-sensitivity, making it difficult to distinguish between strain and temperature from single Bragg wavelength shifts. To address this issue, Sanjib Sarkar employed a multi-target supervised ensemble regression algorithm from machine learning to simultaneously predict strain and temperature. By learning the relationship between the reflected spectrum and its corresponding temperature and strain, the Boosting ensemble estimator effectively separated temperature from strain. The study compared two averaging ensemble methods—random forest regression (RFR) and Bagging regression (BR)—with two boosting ensemble methods—gradient-boosting regression (GBR) and adaptive boosting regression (ABR), finding GBR to perform the best, with post-separation errors for temperature and strain within 10% of actual values [51,52]. The Extreme Learning Machine (ELM) was also applied to quickly and accurately determine strain and temperature from fiber optic sensors, which exhibit central wavelength shifts due to changes in strain, temperature, grating period, and refractive index. Using ELM to analyze the spectrum alongside temperature and strain data from two sensors facilitated the discernment of their interrelationships. When compared with centroid, Gaussian polynomial fit, and back propagation algorithms, ELM demonstrated superior precision (RMSE = 0.0906) and response time (t = 0.325) [53].

Distributed Acoustic Sensing (DAS) technology senses sound or vibration by measuring phase changes of light transmitted through a fiber optic. For this sensing technique, X. Dong et al. introduced a novel denoising method based on CNN, termed L-FM-CNN, for processing random and coherent noise in distributed fiber optic acoustic-sensing Vertical Seismic Profile (VSP) data. This method combines leaky rectifier linear unit activation functions, forward modeling, and energy ratio matrix (ERM) to enhance the signal-to-noise ratio (SNR). Experimental results showed an SNR improvement of over 10 db using L-FM-CNN compared to methods like DnCNNs [54].

Instrumental variation poses significant challenges in the sensor field due to differences in sensor and device manufacturing that result in varied responses to identical signal sources and time-varying drift, characterized by changes in sensor attributes, operational conditions, or the signal source over time. Models trained on data from an earlier period are not suitable for new devices or data from later periods due to these variations. To overcome these challenges, Ke Yan introduced Maximum Independent Domain Adaptation (MIDA) and a semi-supervised version of MIDA. These methods address instrumental differences and time-varying drift by treating them as discrete and continuous distribution changes in the feature space and then learning a subspace that maximizes independence from the domain features, thereby reducing the discrepancy in distributions across domains. The effectiveness of the proposed algorithms is demonstrated through experiments on synthetic datasets and four real-world datasets related to sensor measurement, significantly enhancing the practicability of sensor systems [55].

In summary, incorporating AI methods into the design process of sensors can streamline design time, reduce computational costs, and minimize iterations, facilitating the rapid development of configurations that meet specific environmental or functional requirements. Moreover, integrating ML/DL algorithms into the signal-processing phase significantly improves critical parameters. Yet, AI’s role in sensor design faces challenges, including the extensive training necessary for AI algorithms to facilitate design. Moreover, previously trained models risk becoming outdated due to the algorithms’ inability to interpret the complex interplay of multi-field responses of devices, rendering them incapable of anticipating performance changes over time, such as aging. This underscores the limitations in the universality of AI-driven sensor design.

## 3. Calibration and Compensation

During their operation, sensors often experience signal drift due to voltage fluctuations, temperature changes, or other environmental factors, leading to distorted measurement results. To address this issue, ML/DL algorithms employ two strategies: Firstly, algorithms such as ELM and MLP are used during calibration to consider the effects of various environmental factors, reducing the need for repetitive calibration tests, decreasing calibration time, and enhancing precision. Secondly, algorithms like MLP and CNN are introduced during usage to automatically compensate for various disturbances encountered in the environment.

### 3.1. Pre-Use Calibration

Due to the electrical properties of sensor elements changing with temperature and the sensitivity of the units themselves to temperature variations, pressure sensor electrical signals can be significantly impacted by changes in ambient temperature. To address this issue, an automatic calibration algorithm for capacitive pressure sensors, based on rough set neural networks (RSNNs), was proposed. This algorithm models the sensor’s response characteristics using rough set theory and calibrates the sensor’s nonlinear response to temperature changes using neural networks, effectively mapping the sensor response curves across various environmental temperatures. The model estimates pressure with an accuracy of ±2.5% (FS) across a temperature range of −50 °C to 150 °C [56]. Similarly, the MLP algorithm has been utilized for calibration assistance, accurately estimating pressure with an error margin of ±1% (FS) within the same temperature range [57].

In the calibration of sensors, artificial intelligence algorithms not only reduce signal drift caused by environmental factors but also decrease the workload associated with calibrating nonlinear sensor response curves. For example, for nonlinear temperature sensors, José Rivera developed an automatic calibration method based on ANN. The study analyzed various network topologies like MLP and radial basis function (RBF), along with training algorithms such as backpropagation, the conjugate gradient algorithm, and the Levenberg–Marquardt algorithm. They found these methods offer superior overall accuracy compared to piecewise linearization and polynomial linearization methods, enabling intelligent sensors to be calibrated more quickly and accurately, addressing issues like offset, gain changes, and non-linearity. With five calibration points, the error rate was 0.17%, and the calibration time was reduced to 3523 ms for five to eight calibration points [58]. For pressure sensors, an ELM-based method was applied, utilizing ELM’s capability to approximate any nonlinear function, calibrating system errors caused by temperature and voltage fluctuations. The ELM showed optimal performance in both calibration accuracy and speed, with an RMSE of 0.546 and a calibration time of 1.3 s [59]. Expanding to broader sensor calibration types, Alessandro Depari et al. introduced a two-stage method based on the Adaptive Network-based Fuzzy Inference System (ANFIS), requiring fewer calibration points and lower computational power during the recalibration phase. The first stage involves preliminary calibration of the sensor system under standard conditions using a large number of calibration points to train the ANFIS. The second stage, requiring relatively fewer points and parameter adjustments through gradient descent, facilitates recalibration, reducing computational effort and enabling online recalibration. This method, applied in a pyroelectric biaxial positioning system, achieves a resolution of 20 μm across the entire 7 mm × 7 mm detectable area [60].

### 3.2. In-Use Calibration

**Figure 2:**
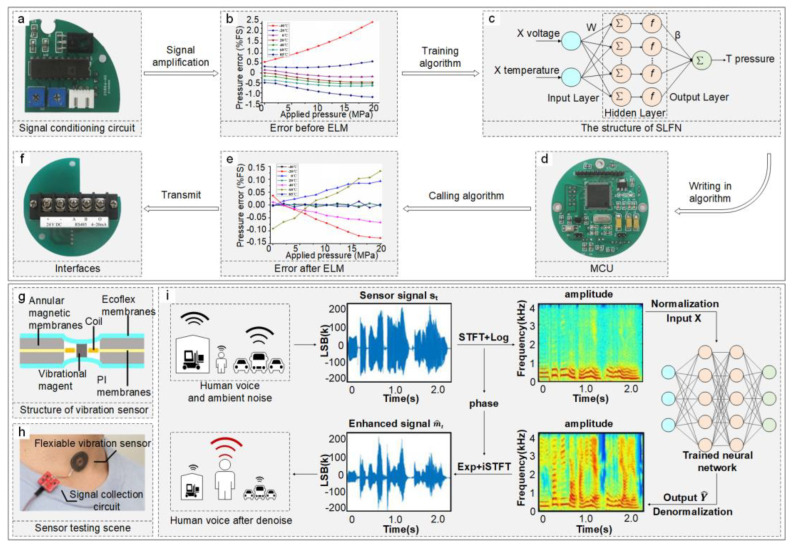
Application of ML/DL algorithms in calibration and compensation. (**a**–**f**) Process of utilizing an ML algorithm for compensating sensor thermal drift: (**a**) signal conditioning circuit for data normalization. Adapted with permission. Copyright 2014, Multidisciplinary Digital Publishing Institute [61]. (**b**) Calculation of pressure error prior to temperature compensation. Reproduced with permission. Copyright 2014, Multidisciplinary Digital Publishing Institute [61]. (**c**) Configuration of the SLFN trained with testing data. Adapted with permission. Copyright 2014, Multidisciplinary Digital Publishing Institute [61]. (**d**) MCU with digital thermometer and controller chip for storing SLFN weights and biases. Adapted with permission. Copyright 2014, Multidisciplinary Digital Publishing Institute [61]. (**e**) Pressure error following temperature compensation. Reproduced with permission. Copyright 2014, Multidisciplinary Digital Publishing Institute [61]. (**f**) Interface circuit for digital signal output. Adapted with permission. Copyright 2014, Multidisciplinary Digital Publishing Institute [61]. (**g**–**i**) Process of using a DL algorithm for speech enhancement: (**g**) flexible vibration sensor placed on a volunteer’s neck. Reproduced with permission. Copyright 2022, American Institute of Physics [68]. (**h**) Cross-sectional view of the vibration sensor. Reproduced with permission. Copyright 2022, American Institute of Physics [68]. (**i**) Neural network-based algorithm flow for ambient noise reduction, including signal collection, speech decomposition, feature extraction and enhancement. Adapted with permission. Copyright 2022, American Institute of Physics [68].

Temperature significantly influences pressure sensor signals, necessitating compensation for temperature-induced errors to enhance sensor accuracy, an important application for ML/DL algorithms. A typical compensation process involves the following [61]:

1. Test devices within specified temperature and pressure ranges. Data are conditioned by a signal conditioning circuit [Figure 2a], normalized to the range of [−1, 1], and measurement error is calculated [Figure 2b].
2. Divide the normalized sample data (voltage, temperature, applied pressure) randomly into training and testing datasets at a 2:1 ratio.
3. Sequentially choose the number of hidden nodes, starting from one up to the number of training samples.
4. Initialize input weights and hidden layer biases, then compute the Single-Layer Feedforward Neural Network’s (SLFN) output weights using the training data.
5. Utilize the weights and biases obtained in Step 4 to compute the output for the testing data.
6. Repeat Steps 2 to 4 until achieving satisfactory compensation accuracy [Figure 2c].
7. Program the SLFN weights and biases into a micro-control unit (MCU) equipped with a digital thermometer and chip [Figure 2d] and validate the algorithm within the defined temperature and pressure ranges.
8. Implement automatic temperature error compensation using the algorithm [Figure 2e], employing an interface circuit [Figure 2f] for digital signal output and communication with a PC.

Diverse automatic compensation techniques have been developed for various pressure sensors by adapting the outlined steps. Notably, an Artificial Neural Network (ANN) strategy provided intelligent temperature compensation for porous silicon micro-machined piezoresistive sensors, achieving temperature-independent outputs by minimizing sensor bias voltage fluctuations due to temperature changes. This approach involved modeling the sensor’s operational range, creating an inverse model by connecting the sensor to an ANN, and training the ANN to adapt to temperature shifts, ultimately reducing uncompensated temperature error by about 98% [62]. Further, ANNs employing conventional and inverse delayed function neuron models significantly reduced temperature drift errors in high-resolution sensors [63]. For extreme conditions, an MLP-based model yielded automatic compensation with an accuracy within ±0.5% across a broad temperature range. Similar methods enhanced fiber optic sensors’ accuracy to over 95% by compensating for temperature-related expansion and bending losses [64]. Guanwu Zhou used 88 sets of temperature and pressure combinations as learning samples (training set to validation set ratio of 2:1) to compare the calibration performance of various methods, including VCR, RPA, BP, SVM, RBF, and ELM. The results indicated that, compared with other algorithms, ELM exhibited superior generalization capabilities and faster learning speeds even with a smaller calibration sample size. ELM achieved higher accuracy (0.23%) and was capable of calibrating pressures ranging from 0 to 20 MPa within a temperature span of −40 °C to 85 °C [61].

Aside from temperature-related inaccuracies, sensor errors can stem from various factors like noise from power supply or semiconductor signal interference, fixed offsets due to manufacturing flaws, temperature shifts or other environmental influences, and drifts where the sensor output-to-input ratio changes over time. These combined factors can gradually diminish the accuracy of MEMS-based Inertial Navigation Systems. Hua Chen devised a CNN-based approach to mitigate these disturbances in inertial sensor signals. This method processes Inertial Measurement Unit (IMU) raw data, including errors, through CNNs that segment data into smaller time units for error identification, achieving an 80% accuracy in distinguishing accelerometer and gyroscope signals compared to traditional static and rate tests [65]. Furthermore, an automatic compensation method using FLANN addresses changes in the pressure sensor environment, manufacturing parameter shifts, and aging effects, maintaining maximum error within ±2% [44]. In gas sensors, aging (e.g., surface reorganization over time) and poisoning (e.g., irreversible binding from contamination) also pose challenges due to physical and chemical reactions between chemical analytes and the sensor film in the gas phase. Alexander Vergara proposed an ensemble method using a weighted combination of classifiers trained at different times with Support Vector Machines (SVMs) to mitigate such effects. This approach updates classifier weights based on their current batch performance, allowing for drift identification and compensation and enhancing gas recognition accuracy post-drift to 91.84% [40].

Additionally, specific scenarios, like uneven pressure distribution and insufficient curvature adaptation in robotic arm applications, can cause sensor drift. Dong-Eon Kim established lookup tables to linearize outputs from resistive barometric sensors based on cubic weight, employing linear regression, lookup methods, and supervised learning with known object weights as training data to ensure stable grip force measurement [66].

In acoustical signal processing scenarios, voice enhancement serves as a specific form of sensor signal compensation, addressing the issue of consonant phoneme loss due to high-frequency attenuation in traditional throat microphones. Shenghan Gao and his team developed a flexible vibration sensor based on non-contact electromagnetic coupling [Figure 2g] for capturing vocal fold vibration signals [Figure 2h]. They utilized short-time Fourier transform (STFT) to decompose speech into amplitude and phase, employing four neural network models—fully connected neural network (FCNN), long short-term memory (LSTM), bidirectional long short-term memory (BLSTM), and convolutional-recurrent neural network (CRNN)—to extract and enhance speech data features [Figure 2i]. Experimental results indicated that BLSTM performed best in improving speech quality but was the least favorable for hardware deployment, boosting short-time objective intelligibility (STOI) from 0.18 to nearly 0.80 [67].

Overall, AI algorithms can reduce errors caused by environmental changes, voltage fluctuations, and other factors during sensor calibration or automatically compensate for errors due to environmental changes, voltage fluctuations, and device aging during sensor use. However, the application of AI in calibration and automatic compensation faces challenges. ML/DL models, being black-box in nature, make it difficult to quantitatively explain the proportion of various factors contributing to device drift, limiting guidance for sensor design improvements. Furthermore, ML/DL model training typically requires extensive data and computational resources, and the models’ limited generalizability can result in poor performance in new environments.

## 4. Recognition and Classification

In sensor applications, artificial intelligence (AI) extends beyond mere signal collection to enable the identification and classification of objects and application scenarios. The AI-assisted recognition and classification process typically involves data collection by sensors, feature extraction, feature matching, and making identifications. By employing algorithms such as random forest (RF), KNN, SVM, and Deep Belief Networks (DBNs), it is possible to reduce decision-making time in recognition, increase accuracy, lower the cost of manual identification, and minimize environmental interference for more precise feature extraction. The complexity of the application scenarios dictates the sensor information requirements; for instance, voice recognition can be achieved solely through vibration signals, whereas motion recognition often necessitates a combination of signals from visual and pressure sensors.

### 4.1. Classification and Recognition Based on Unidimensional Data

Applications of ML/DL-based recognition and classification in sensors span a broad spectrum, including robotic perception, object identification, behavior recognition, health monitoring, identity verification, and mechanical fault detection.

#### 4.1.1. Robotic Perception

In robotics, ML/DL algorithms are extensively applied in gesture recognition [68], full-body motion detection [69], and tactile sensing [70,71,72,73]. For instance, a robotic fingertip tactile sensor based on imaging and shallow neural networks can detect the location and local curvature radius (ROC) of object contacts while measuring force. Made of silicone resin with internal markers, its geometry and physical properties are learned through camera-captured marker displacements. Researchers developed a hierarchical neural network, including Contact Pos Net for estimating contact positions and forces and Classification Net for surface type categorization. Utilizing 72 input features of marker displacements, the trained model achieves identification/measurement errors as low as 1 mm for contact position and 0.3 N for force [74]. Moreover, soft magnetic skin, combined with machine learning, detects continuous deformation and identifies deformation sites. This skin, consisting of a magnetometer, silicone rubber, and magnetic particles, alters the surrounding magnetic field’s intensity when compressed or deformed, allowing deformation site identification with up to 98% accuracy using Quadratic Discriminant Analysis (QDA) [75].

In tactile recognition, transforming pressure distributions into cloud diagrams for object recognition via image analysis offers another method [38]. Juan M. Gandarias discussed two AI approaches for object identification using high-resolution tactile sensor pressure images: one uses Speeded-Up Robust Features (SURFs) for feature extraction, and the other employs a Deep Convolutional Neural Network (DCNN). The features are then classified: the first method clusters features into a dictionary using k-means to create a Bag of Words (BoWs) framework, while the second uses a pre-trained network for conventional image classification followed by supervised SVMs for identifying object shapes and textures. Experiments showed that SURF-based feature extraction is five times faster than DCNN, though DCNN-based feature extraction achieved an accuracy 11.67% higher than SURF [76]. Furthermore, the precision of such tactile recognition techniques has been systematically evaluated. CNN models pre-trained on one million images (SqueezeNet, AlexNet, ResNet, and VGG16) were adapted through transfer learning for use on a pressure cloud dataset, followed by classification with fully connected layers and SVM classifiers to identify contact objects. As a comparison, a custom-built CNN model (TactNet), trained on a dataset of 880 tactile pressure images, was used for object recognition. Results indicated that, with a smaller sample size (880 samples), the pre-trained fully connected layer CNN had the highest recognition accuracy at 95.36%, followed by TactNet at 95.02%, with CNN-SVM performing the least accurately at 93.05%. In terms of recognition speed, TactNet (0.094 s to 0.465 s) was significantly faster than the pre-trained CNN models (4.141 s to 73.355 s) [38].

Besides accuracy, recognition speed is crucial for algorithmic applications in robotic perception. Hongbin Liu introduced a novel rapid computation algorithm that uses a tactile array sensor on robot fingertips for real-time classification of local contact shapes and postures with an NB classifier. By analyzing the covariance between pressure values and their sensor positions, it extracts tactile images with lower structural properties and determines three orthogonal principal axes for contact shape classification, unaffected by rotation. This approach achieves a total accuracy rate of 97.5% for classifying six basic contact shapes and is highly efficient in computation (total classification time for local contact shapes = 576 μs) [77].

#### 4.1.2. Object Identification

ML/DL algorithms assist in identifying solid material types through sensor integration [78]. Nawid Jamali devised a machine learning model that distinguishes materials based on surface texture, using strain gauges and PVDF (Polyvinylidene Fluoride) films embedded in silicone on robotic fingers. Movement across material surfaces generates vibrations in silicone, proportional to movement speed and texture variation, serving as input for algorithms. A majority vote classification method consolidates decisions from trained naive Bayes, decision trees, and naive Bayes trees algorithms, selecting the most voted category. This approach accurately differentiates materials like carpet, vinyl flooring, tile, sponge, wood, and PVC mesh with a 95% ± 4% accuracy rate [79]. Similarly, graphene tactile sensors, combined with KNN algorithms, classify textile materials with up to 97% accuracy [80].

Beyond solids, AI-enhanced sensors can identify components in liquid mixtures. For instance, detecting alcohol in water through light intensity changes in optical fiber sensors, where neural networks enhance identification accuracy. The process involves using OTDR to collect light intensity data from optical fiber sensors under various conditions (e.g., air, water, alcohol), followed by training a three-layer feedforward neural network to recognize the presence of alcohol based on the light intensity data. This trained network accurately predicts alcohol presence in new samples [81].

Additionally, sensor–ML/DL combinations are extensively applied in gas detection [82,83]. Bionic olfactory sensors paired with CNNs can identify toxic indoor gas components. Utilizing an electronic nose equipped with ten cross-sensitive metal oxide gas sensors, odor data are collected and processed into a dataset comprising training and testing sets with 728 and 312 samples, respectively, each featuring 600 distinct characteristics. This setup enables the plotting of response curves for different toxic gases within the same interference group. CNNs are then employed to analyze electronic nose data to identify harmful gases (formaldehyde, ammonia, benzene, methanol) in mixtures, reaching a 90.96% accuracy rate [84]. Mixed gas detection is crucial for safety and production sectors.

#### 4.1.3. Human Behavior Recognition

In human posture recognition, sensors often need to adhere to the skin, where skin curvature changes significantly from the flat state during sensor calibration. Machine learning aids in compensating for curvature-induced drifts, enhancing recognition accuracy [85,86,87]. For instance, a six-axis Force-Sensitive Resistor (FSR) sensor, combined with KNN, can classify and recognize human motions with an accuracy of 86% and a training time of only 25.1 s [88]. Accelerometers combined with SVM algorithms can identify movement patterns and abnormal gait cycles, achieving 83% accuracy and providing crucial data for clinical treatments [89]. The choice of algorithm is critical for accurately detecting abnormal gait [90], as different algorithms yield varying results.

Moreover, ML/DL algorithms often pair with wearable wireless devices, enabling real-time recognition of outdoor activities [29,91]. However, the complexity of outdoor environments necessitates machine learning to compensate for environmental drifts [92,93]. Neelakandan Mani introduced a smart sling with strain sensors and machine learning to monitor human activities. The smart sling’s strain sensors collect strain data during activities, with features reflective of human motion extracted using the Kernel Density Approach (KDA). These features are then classified using an LSTM-based algorithm to identify specific activities (walking, running, sitting, standing, eating, writing), achieving an accuracy of 97.85% [94]. Integrating flexible full-textile pressure sensors with CNNs allows for the recognition of human activities. By monitoring muscle motion during dumbbell exercises, the sensor accurately collects stable, repeatable pressure signals. A trained CNN analyzes characteristic peaks in the response current curve to distinguish subtle muscle movement changes, achieving a 93.61% identification accuracy [95].

**Figure 3:**
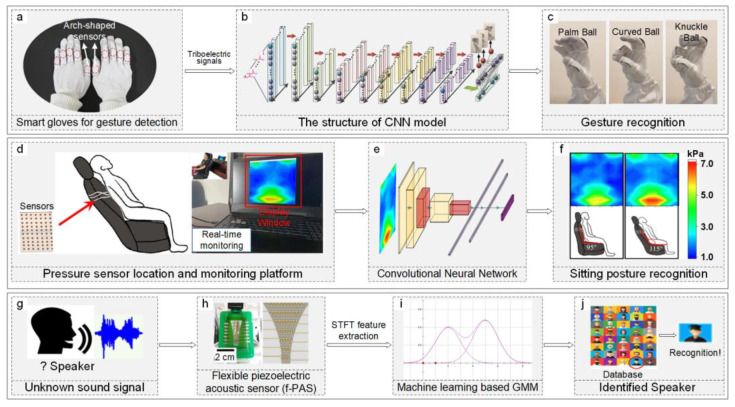
Application of ML/DL algorithms in recognition and classification using unidimensional data. (**a**–**c**) Illustration of gesture recognition through a DL algorithm. (**a**) Depiction of smart gloves with embedded strain sensors for data acquisition. Reproduced with permission. Copyright 2020, WILEY-VCH Verlag GmbH & Co. KGaA, Weinheim, Germany [27]. (**b**) Diagram of a CNN model refined using testing data. Adapted with permission. Copyright 2020, WILEY-VCH Verlag GmbH & Co. KGaA, Weinheim, Germany [27]. (**c**) Classification of three distinct gestures based on strain data. Adapted with permission. Copyright 2020, WILEY-VCH Verlag GmbH & Co. KGaA, Weinheim, Germany [27]. (**d**–**f**) Procedure of recognizing sitting posture inclination with a DL algorithm. (**d**) Display of a strain sensing array on a seat backrest (left) and the corresponding data acquisition and visualization system (right). Reproduced with permission. Copyright 2022, Institute of Physics [105]. (**e**) Outline of a CNN framework adjusted with testing data. Reproduced with permission. Copyright 2022, Institute of Physics [105]. (**f**) Visualization of pressure contours and their associated sitting posture recognitions. Adapted with permission. Copyright 2022, Institute of Physics [105]. (**g**–**j**) Method of speech recognition via an ML algorithm. (**g**) Vocal signal from an unidentified speaker. ELSEVIER. Adapted with permission. Copyright 2018, ELSEVIER [115]. (**h**) Visuals of an f-PAS for vocal signal capture (left) alongside a schematic of a f-PAS (right). Reproduced with permission. Copyright 2018, ELSEVIER [115]. (**i**) Conceptual diagram of a GMM refined with testing data. (**j**) Speaker search and identification within a database. Adapted with permission. Copyright 2018, ELSEVIER [115].

Joint or muscle movements often induce subtle yet distinct strains on the skin, making strain sensors highly sensitive to variations in motion. For example, knee joint movements can be detected using a wearable system based on strain sensors. Neural networks and RF algorithms used to analyze knee joint angles during walking and static bending tasks show mean absolute errors (MAEs) of 1.94 degrees and 3.02 degrees, respectively, with a coefficient of determination (R^2^) of 0.97. This method proves more accurate than traditional linear approaches, improving precision by about 6 degrees [96]. Finger joint movements can be recognized in real-time by integrating carbon nanotube (CNT)-based resistive strain sensors into a textile glove [Figure 3a]. The resistance changes in the CNTs/TPE coating due to strain are converted into electrical signals, then analyzed and learned using CNNs [Figure 3b] to identify different hand gestures or joint motion patterns [Figure 3c], achieving a 99.167% recognition accuracy for precise VR/AR control, including shooting, baseball pitching, and flower arranging [27]. Furthermore, gesture recognition extended to sign language interpretation using algorithms like SVM boasts up to 98.63% accuracy and less than one second recognition time [97].

Recognizing joint or muscle movements typically involves categorizing by electrical signal strength or phase differences. Due to the frequent changes in joint or muscle movement, monitoring data through bioelectrical and triboelectric signals is a common method, where machine learning significantly improves recognition accuracy [42]. Bioelectric sensors, which convert biological reactions into measurable electrical signals, facilitate the recognition of human gestures in handball games. Various gestures, which trigger muscle group signals, are captured by the Myo armband gesture control. Data from eight bioelectrical potential channels for each gesture by two players are collected, creating a dataset that, after preprocessing and feature extraction, is trained using an SVM model to distinguish five different gestures, achieving recognition accuracies of 92% and 84% for the two players [98].

Posture recognition in human behavior is a common application [99,100,101,102], significantly relevant for monitoring systems and security analysis. AI-assisted sensors can provide real-time posture alerts [103], reducing the need for manual care. For instance, the LifeChair smart cushion, incorporating pressure sensors, a smartphone app interface, and machine learning, offers real-time posture recognition and guidance. The posture dataset comprises user BMI, timestamps, raw sensor values, and posture labels, with the RF algorithm learning the mappings between raw sensor values and postures for recognition. It achieves high recognition accuracy, up to 98.93% [104], for over thirteen different sitting postures. Additionally, human posture inclination can be identified by combining flexible pressure sensors and neural networks. Initially, large-area flexible pressure sensors collect data from the human back [Figure 3d]; these pressure data are then input into a pre-trained neural network [Figure 3e] that determines the body’s inclination based on the input pressure data [Figure 3f], with recognition accuracies ranging from 0.94 to 0.98 for five postures [105].

To enhance the accuracy of sitting posture recognition, Jongryun Roh compared the efficacy of multiple algorithms within a low-complexity posture monitoring system that employs four pressure sensors mounted on a seat. These sensors collect data related to user weight and the orientation of the sitting posture, both front-to-back and side-to-side. Various machine learning algorithms, including SVM with RBF kernel, LDA, QDA, NB, and a random forest classifier, were applied to classify six sitting postures using 84 posture samples. The decision tree showed the lowest accuracy at 76.79%, while the SVM with RBF kernel achieved the highest at 97.2% [93]. In addition to accuracy, model training time is a critical metric for sensor recognition algorithms. Aurora Polo Rodríguez proposed a method using Pressure Mat Sensors to classify human postures in bed. They transformed raw pressure data into grayscale visualizations for analysis, collected 232 samples, and utilized data augmentation techniques to expand the dataset by generating synthetic sleeping postures. By comparing two CNN models with different numbers of convolutional layers and stages of dropout layer usage, both models reached accuracies of 98%, with the model having fewer convolutional layers requiring only two-thirds the training time of the more complex model [103].

Beyond activity, human rest also requires monitoring and feedback, as analyzing sleep behavior can improve sleep issues. Carter Green et al. developed a TCN model trained with data from an under-bed pressure sensor array to recognize and classify sleep postures and states. Information related to sleep, such as event types, start times, and durations, was extracted from polysomnography (PSG) and pressure sensor mat (PSM) data. Features extracted from PSM data, including the number of active sensors, the sum of weighted sensor values, lateral center pressure, lateral variance, and longitudinal center pressure, served as inputs for a CNN, with body position (supine, prone, left side, right side) and a Boolean value of sleep state as outputs. With data augmentation, a classification accuracy of 0.998 was reported [106]. This tool, as an economical and effective sleep assessment method, holds great potential, simultaneously reducing patient burden and professional workload.

#### 4.1.4. Health Monitoring

In the domain of human health monitoring, sensors often measure vital information such as blood pressure and heart rhythm, which are then analyzed by AI algorithms to diagnose potential diseases [107]. Sun Hwa Kwon developed a method for detecting cardiac abnormalities using machine learning. In this approach, flexible sensors attached to the chest collect electrocardiogram (ECG) signals through piezoelectric or triboelectric effects, translating them into signals like heart rate, blood pressure, and respiratory rate. CNN algorithms are then applied to extract features and classify the data, achieving a recognition accuracy of 98.7% for cardiac abnormalities [33]. Another example involves an embedded system integrating TinyML and an electronic nose equipped with metal oxide semiconductor (MOS) sensors for real-time diabetes detection. Researchers collected exhaled gases from 44 subjects (comprising 22 healthy individuals and 22 diagnosed with various types of diabetes mellitus), transferred them to the sample chamber of the electronic nose, and collected sensor data via a microcontroller. After data preprocessing and feature selection, selected features were used to train machine learning models, such as XGBoost, DNN, and one-dimensional convolutional neural networks (1D-CNN). Finally, real-time samples of exhaled gases were collected by the electronic nose system, and the integrated TinyML model was used to determine if the subjects had diabetes. Among these, the XGBoost machine learning algorithm achieved a detection accuracy of 95%, DNN achieved 94.44%, and 1D-CNN achieved 94.4% [108].

Additionally, pulse signals can be utilized for health monitoring. Yunsheng Fang developed a cost-effective, lightweight, and mechanically durable textile triboelectric sensor. This sensor converts minute skin deformations caused by arterial pulsation into electrical energy, enabling high-fidelity and continuous monitoring of pulse waveforms even in mobile and sweating conditions. Employing a supervised feedforward neural network architecture, the model automatically extracts pulse features, providing continuous and precise measurements of systolic and diastolic pressures with average deviations of 2.9% and 1.2%, respectively [32].

On the other hand, Michael Roberts et al. analyzed the accuracy of AI algorithms in disease detection. They summarized research utilizing ML/DL to detect and predict COVID-19 from standard-of-care chest radiographs (CXR) and chest computed tomography (CT) images. The majority of these studies utilized CNN or deep learning models for feature extraction, while a minority combined hand-engineered radiomic features and clinical data. These studies trained and tested models on collected samples, with the majority achieving recognition accuracies of 85% or higher, while a few reached around 70%. However, due to methodological flaws and/or potential biases, the identified models lack clinical applicability. Reasons for this include biased small datasets, variability in large international dataset sources, and poor integration of multi-stream data, especially imaging datasets [109].

#### 4.1.5. Identity Verification

Sensors combined with ML/DL algorithms can identify individuals by recognizing behavioral patterns [110,111,112,113]. Qiongfeng Shi developed self-powered triboelectric floor mats to capture pressure signals from footsteps in real-time. These signals are analyzed using a pre-trained CNN model to identify individuals based on learned user characteristics, such as verifying if they are authorized room users. This identification controls lighting and door access, enabling smart indoor environment management [114].

Identity recognition can also be achieved through voice recognition. Voice vibrations through a piezoelectric film generate voltage signals due to the piezoelectric effect. Integrating piezoelectric sensors with machine learning creates a system for recognizing speakers. The system captures vocal signals [Figure 3g] with flexible piezoelectric acoustic sensors (f-PAS) [Figure 3h], processes these signals through filtering, amplification, and digital conversion, and extracts vocal features [Figure 3i]. A trained Gaussian Mixture Model (GMM) algorithm then identifies the speaker with 97.5% accuracy [Figure 3j] [115]. Similarly, deep learning models like DNNs, CNNs, or RNNs, trained on voice signal features such as mel-frequency cepstral coefficients (MFCCs) or spectrograms and corresponding labels (speaker identity), achieve over 90% accuracy in speaker identification [28]. Additionally, Optical Microfiber sensors can be applied to the larynx to monitor vocal cord vibrations, enabling speech recognition through the utilization of a 1D-CNN algorithm, achieving an accuracy of up to 89% [116].

#### 4.1.6. Mechanical Fault Detection

In mechanical fault identification, sensors combined with ML/DL algorithms can monitor the operational status of machinery in real-time [30], promptly identify anomalies, and facilitate repairs before issues escalate. This reduces downtime and enhances productivity. Vibration sensors are crucial for this task due to the significant role of vibrations in mechanical operations. Chuan Li and colleagues developed a deep statistical feature learning method for diagnosing faults in rotating machinery by extracting temporal, frequency and time-frequency domain features from vibration sensor signals to generate a statistical feature set. They employed a Gaussian-Bernoulli Deep Boltzmann Machine (GDBM) for automated learning of fault-sensitive features. The model was pre-trained using an unsupervised learning algorithm, fine-tuned with the backpropagation (BP) algorithm, and applied to diagnose faults in gearboxes and bearing systems with accuracies of 95.17% and 91.75%, respectively [117]. Jie Tao introduced a bearing fault diagnosis method using a Deep Belief Network (DBN) and information fusion from multiple vibration sensors. This method extracts vibration signals and temporal domain features from different faulty bearings, adaptively fusing multi-feature data using the DBN’s learning capability. Fault diagnosis is completed by inputting data from multiple sensors into the DBN to generate a classifier, achieving an identification accuracy of 97.5% for inner race, outer race, and ball faults [118].

Tire pressure loss, a common vehicle issue, poses a risk to road safety. Lingtao Wei proposed a machine learning-based, low-cost framework for detecting tire pressure loss, addressing the high costs, lack of redundancy, and dependence on the proper functioning of pressure sensors in existing monitoring methods. The strategy involves feature extraction employing a rigid tire model, correction of manufacturing inaccuracies in speed gears via the recursive least square method, and velocity measurement based on intervals captured by wheel speed sensors. Additionally, it encompasses the extraction of both time- and frequency-domain features from velocity signals. Finally, the tire pressure status is accurately determined using Support Vector Machine (SVM) analysis after DT filtering, achieving a precision rate of 96.18% [31].

### 4.2. Classification and Recognition Based on Multi-Dimensional Data

In practical applications such as human behavior recognition, object identification, or fault monitoring, relying solely on single-type signal data for analysis might lead to issues with accuracy or limited applicability. Utilizing artificial intelligence to fuse and analyze data from sensors capturing various signal types can enhance recognition accuracy.

#### 4.2.1. Human Behavior Recognition

**Figure 4:**
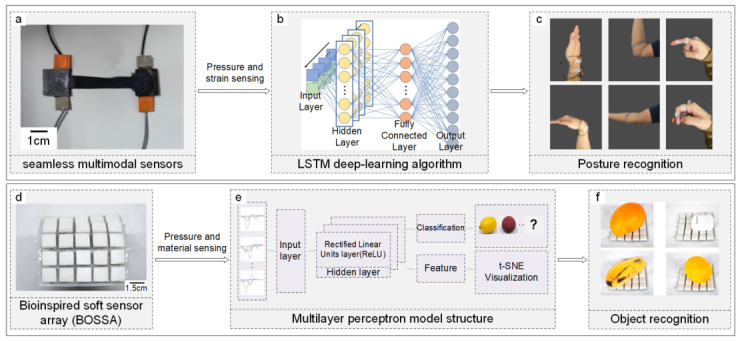
Application of ML/DL algorithms in recognition and classification using multi-dimensional data. (**a**–**c**) Process of recognizing joint movement states with a DL algorithm. (**a**) Depiction of seamless multimodal sensors designed for pressure and strain data gathering. Adapted with permission. Copyright 2022, Nature Publishing Group [121]. (**b**) Schematic of an LSTM network refined with testing data. Adapted with permission. Copyright 2022, Nature Publishing Group [121]. (**c**) Recognition of six joint movements based on pressure and strain measurements. Adapted with permission. Copyright 2022, Nature Publishing Group [121]. (**d**–**f**) Demonstration of object recognition via a DL algorithm. (**d**) Optical image of the 5 × 5-pixel BOSSA sensor array for acquiring pressure and material data. Reproduced with permission. Copyright 2022, American Chemical Society [126]. (**e**) Structure of an MLP model optimized with testing data. Adapted with permission. Copyright 2022, American Chemical Society [126]. (**f**) Identification of objects using pressure and material information. Adapted with permission. Copyright 2022, American Chemical Society [126].

Research on human behavior recognition and classification has primarily focused on significant movements like overall body motion or muscle and joint movements. A method using Pyroelectric Infrared (PIR) sensors, which detect infrared heat from human or animal activity, has been applied for human motion detection and recognition. The process involves collecting motion data with sensors like PIR and cameras, processing this data to extract features such as signal amplitude and duration from PIR sensor outputs, and identifying movement direction using peak detection methods. Features critical for motion detection, like the three peak values of a PIR signal, are selected and used with classification algorithms like SVM and KNN to recognize motions, achieving over 94% accuracy in identifying walking direction and speed [119]. For research recognizing muscle or joint movements, wearable sensors offer a simple yet effective solution. Jianhui Gao et al. applied resistance/capacitance dual-mode (RCDM) sensors to measure joint compression and stretchable strain during tennis, using LSTM networks to accurately identify joint movements with a 97.21% recognition rate [120]. Additionally, wearable seamless multimodal sensors [Figure 4a] can decouple pressure and strain stimuli and, with LSTM deep learning algorithms [Figure 4b], recognize different joint movement states with a 97.13% accuracy rate [Figure 4c], demonstrating the capability to differentiate joint positions and states with the assistance of machine learning algorithms [121].

Beyond studying substantial human movements like motion, some research also focuses on subtle activities such as swallowing and breathing. For instance, Beril Polat used Epidermal Graphene Sensors to measure strain and sEMG signals, employing machine learning to estimate the volume of swallowed water and distinguish between actual swallowing actions and motion artifacts. Using SVM algorithms, the cumulative volume of swallowed water from 5 to 30 mL was estimated, with an accuracy rate exceeding 92% [122]. Ke Yan et al. explored feature selection in gas detection to assist in diabetes screening by analyzing gas components in breath samples. Initially, gases were collected using Carbon Dioxide Sensors, Temperature-Humidity Sensors, and Metal Oxide Semiconductor Sensors in an electronic nose. They optimized feature selection with Support Vector Machine Recursive Feature Elimination (SVM-RFE) and Correlation Bias Reduction (CBR), effectively distinguishing between healthy subjects and diabetes patients by VOC concentrations. This methodology achieved diabetes detection accuracies of 90.37%, enhanced to 91.67% with CBR, and reached a peak accuracy of 95% when combining nonlinear SVM-RFE with advanced strategies [34].

#### 4.2.2. Object Identification

Object surface texture recognition is a popular research area. When sensors touch an object’s surface, texture judgment is influenced by pressure, sliding speed, acceleration, and position. Multi-sensor data fusion, therefore, aids in more accurately identifying object surface textures. Hideaki Orii utilized pressure and six-axis acceleration sensors, combined with CNN, for tactile texture recognition. After normalizing and denoising the collected data, it was processed as image data through CNN’s convolutional layers to extract temporal features, followed by supervised learning for network parameter training. The difference between network output and expected output updated the weighted value matrix and bias vector of each layer. New input data through the neural network estimated the object category (table, cardboard, non-woven fabric, paper) with an accuracy range of 58.4–94.4% [123]. Similarly, Satoshi Tsuji proposed using CNNs to identify object surface roughness with a simple sensor system composed of a pressure sensor and six-axis acceleration sensors. Measuring time series data—pressure, speed, and posture as the sensor contacts and moves across an object—CNN calculated surface roughness with 71% accuracy [124]. Vibration signals during touch also contribute to texture identification; deep learning techniques extract and classify features from tactile sensor output signals, with some sensors sensitive to static pressure and others to initial contact vibrations. This approach achieved a 99.1% accuracy in texture recognition [36].

Moreover, combining tactile with visual information can enhance texture recognition. Thomas George Thuruthel introduced a system combining multimodal sensors and deep learning for manipulable object recognition and modeling. Tactile information from embedded soft strain sensors and visual information from cameras with autoencoders compressing image data into a low-dimensional feature space enables unsupervised object recognition with an MSE of around 0.002 [125]. Following material determination through texture recognition, object classification can be further refined. Yang Luo developed a bioinspired soft sensor array (BOSSA) [Figure 4d] based on the triboelectric effect, integrating pressure and material sensors within cascaded row and column electrodes embedded in low-modulus porous silicone rubber. This arrangement allows for the extraction of pressure and material information from tactile signals, which is then analyzed using an MLP algorithm [Figure 4e] to identify objects [Figure 4f] by extracting higher-level features. The BOSSA achieves a 98.6% accuracy rate in identifying the types and quantities of ten different objects [126].

Beyond texture recognition, multidimensional data analysis plays a crucial role in robotics research, particularly in differentiating the deformation states of soft actuators crucial for robot control. Two hydrogel sensors detect temperature and various mechanical deformations of soft actuators utilizing a data-driven machine learning approach, such as lateral strain, torsion, and bending. A machine learning model combining 1D-CNN layers with a feed-forward neural network (FNN) decodes sensor signals to identify five states of a soft finger (free bending, bending on contact with room temperature, high-temperature objects, twisting, and stretching), achieving an accuracy of approximately 86.3% [127].

Electronic skin, a significant component in robotics, benefits from multi-signal data fusion. Kee-Sun Soh developed macroscopic electronic skin using a single-layer piezoresistive MWCNT-PDMS composite film equipped with strain and location sensors. A DNN processes resistance changes caused by applied pressure, assessing pressure levels and locations in real-time with over 99% accuracy [128]. Additionally, tactile sensors are employed in electronic skin for object recognition, involving a sensor on a robotic arm touching various objects multiple times at different locations to gather shape information through pressure, surface normals, and curvature measurements. Local features, invariant to translation and rotation, are extracted via unsupervised learning with the k-means algorithm. Object identification then proceeds with a dictionary lookup method, where a dictionary of k words created by k-means facilitates object recognition through a histogram codebook, comparing an object’s histogram distribution to those in a database. This process, requiring only ten touches, achieves a 91.2% accuracy [129].

#### 4.2.3. Mechanical Fault Identification

Monitoring equipment or structural status and analyzing faults are critical for safety assurance. Artificial intelligence, through the fusion and analysis of multi-dimensional data, can more accurately determine equipment states and analyze fault causes. Jinjiang Wang developed a machine learning-based method for estimating tool wear through multisensory data (e.g., force, vibration) analysis, utilizing dimension reduction and support vector regression to measure parameters like tool wear width. The study compared different dimension reduction techniques, including kernel principal component analysis and locally linear embedding, for their efficacy in virtual sensing. The KPCA-SVR model excelled, showing superior performance with a Pearson Correlation Coefficient of 0.9843, RMSE of 5.4428, MAE of 3.9583, and MAPE of 0.037, indicating its effectiveness in tool wear detection [130].

Moreover, large-structure wireless health monitoring can also be achieved through multi-dimensional data analysis. By measuring vibration responses of a cantilever beam with a piezoresistive surface acoustic wave (SAW) accelerometer, exploiting SAW’s modulation by stress/strain during propagation and measuring impedance changes with a pressure sensor, researchers used continuous wavelet transform and Gabor functions for time-frequency analysis of the beam’s free vibration. This allowed for decay coefficient calculation and decay type classification (linear, exponential, or mixed) based on shape changes over time and frequency. They applied three machine learning models, RF, SVM, and LightGBM, to automatically learn decay coefficient features and patterns for damage detection and severity assessment, achieving classification accuracies of 65.4%, 84.6%, and 88.5% on raw data, and 84.6%, 76.9%, and 76.9% on standardized data, respectively [131].

ML/DL-based multi-dimensional data analysis has also been applied to monitor the state-of-charge (SOC) of batteries. Bruno Rente and colleagues developed a SOC estimation method for lithium-ion batteries using FBG sensors and machine learning. FBG sensors monitor the strain and temperature changes during battery usage, indicators closely related to the battery’s internal chemical reactions. Dynamic Time Warping (DTW) standardizes the strain data, which, after being processed with the nearest-neighbor classifier method, accurately estimates the battery’s SOC with an error margin of 2% [132].

In summary, the application of artificial intelligence in recognition and classification enhances accuracy, reduces errors caused by environmental factors, and maintains high response speeds even with complex tasks. However, ML/DL models require substantial amounts of training data, and the limited samples available from sensor data may lead to model overfitting. Additionally, the scarcity of samples complicates the determination of the most suitable model structure, such as the optimal number of layers and parameters.

## 5. Behavior Prediction

Predicting future behavior from data collected by sensors is a crucial application of artificial intelligence in sensing technology. Combining behavior prediction with warning systems can significantly reduce the likelihood of accidents.

In the healthcare and caregiving sectors, timely prediction of patients’ risky behaviors can substantially decrease the chance of injuries and reduce caregiving costs. For patients with severe injuries requiring bed rest, predicting when they might leave the bed becomes crucial. A novel approach utilizing a deep learning model with an 80 × 40 sensor array in bed sheets was developed to monitor sleep posture changes and predict bed-exit behaviors. This method involves collecting sleep pressure data using thin pressure-sensitive sensors and analyzing it with CNNs or Auto Encoders (AEs) to identify sleep postures. The relationship between various sleeping positions and wake-up times was examined to determine which postures predict waking up. With this information, caregivers can take preventive actions, such as providing support or preventing falls before a patient leaves the bed. The prediction accuracy for CNNs reached 92%, while AEs achieved 88% [133].

**Figure 5:**
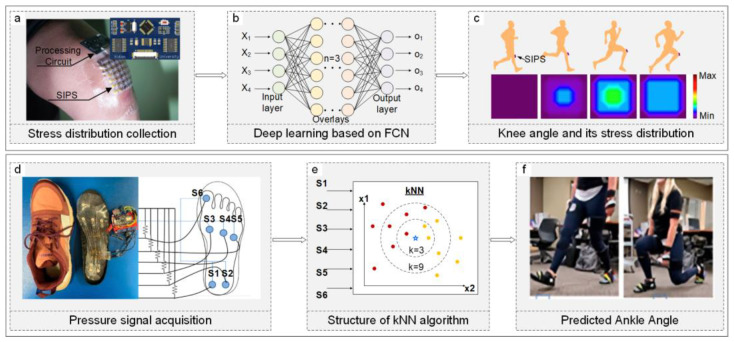
Application of ML/DL algorithms in behavior prediction. (**a**–**c**) Process of knee joint angle prediction via a DL algorithm. (**a**) SIPS with processing circuit on a knee for pressure data collection. Reproduced with permission. Copyright 2022, Nature Publishing Group [133]. (**b**) The structure of FCN was refined with testing data. Adapted with permission. Copyright 2021, Nature Publishing Group [133]. (**c**) Prediction of knee bending states through normalized stress distribution analysis. Reproduced with permission. Copyright 2022, Nature Publishing Group [133]. (**d**–**f**) Process of ankle angle prediction via an ML algorithm. (**d**) Pressure sensor system in insole (left) and schematic overview (right). Adapted with permission. Copyright 2021, Multidisciplinary Digital Publishing Institute [134]. (**e**) Conceptual diagram of KNN algorithm refined with testing data. (**f**) Ankle angle predictions from pressure data. Adapted with permission. Copyright 2021, Multidisciplinary Digital Publishing Institute [134].

Beyond patients with injuries, even those partially recovered need continuous monitoring of their condition to avoid actions that might hinder their rehabilitation. AI-assisted wearable sensor devices can predict and warn against such hazardous behaviors during daily activities. Hongcheng Xu and colleagues developed a stretchable iontronic pressure sensor (SIPS) that senses pressure through changes in the electrochemical double layer (EDL) and electrode contact area [Figure 5a], combined with a fully convolutional network (FCN) algorithm to learn from the collected data [Figure 5b]. This deep learning technique accurately interprets and analyzes complex biophysical signal data from pressure sensors, predicting knee positions from different pressure contours to assess rehabilitation progress and prevent further injury [Figure 5c], with a prediction accuracy of up to 91.8% [18].

Due to the convenience of installing plantar pressure sensors and the ease of data extraction, ML/DL-based predictions are frequently used for foot impact force risk analysis and fall risk prediction. For instance, wearable pressure insoles combined with multiple linear regression (MR) can predict the foot strike angle (FSA), considering factors like weight, height, and age. This process involves collecting running pressure and dynamic data, such as foot landing type and gait pattern, standardizing it, and training the most impactful features on FSA, achieving a prediction accuracy above 90% [134]. Zachary Choffin developed a method using shoe pressure sensors and machine learning to predict ankle angles. Their system [Figure 5d], comprising six force-sensing resistors (FSRs), a microcontroller, and a Bluetooth Low Energy (LE) chip, employs the KNN algorithm to compute the Euclidean distance between training datasets and input data points, identifying the k-nearest data points [Figure 5e]. This method, selecting the ten nearest neighbors, predicts discrete ankle angles with over 93% accuracy during squats and over 87% during bends [Figure 5f] [135]. Additionally, shoe pressure sensors can predict fall risks by collecting dynamic walking data from insoles embedded with wireless pressure sensors, analyzing gait and balance data features, and using logistic regression with oversampling techniques, achieving a high Area Under the Curve (AUC) of 0.88. Furthermore, training with the RF model and oversampling yielded an accuracy of 0.81 and a specificity of 0.88 [136].

In the industrial production field, for safety concerns, such as hazardous gas leaks, the priority is to locate the gas source and address it promptly. Using a convolutional long short-term memory network (CNN-LSTM) to learn from multiple gas sensor fluctuations caused by different gas source locations can quickly identify the gas source under hazardous conditions. This approach accounts for environmental factors like wind direction and speed and changes in the gas source location over time. CNNs clean and extract features from collected data, while LSTMs learn temporal characteristics, and the processed data are input into a DNN to predict the gas source location with an accuracy of 93.9% [137].

Beyond predicting human behaviors, AI-assisted sensor systems are also used to forecast the future states of general objects. For instance, a model combining CNN and bidirectional long short-term memory networks (bidirectional LSTM) is applied to predict actual tool wear. This model initially collects raw sensor data from the tool, including acceleration and sound frequency, to serve as input. A one-dimensional CNN extracts features from the raw input sequence, followed by a two-layer bidirectional LSTM that encodes temporal patterns. On top of the LSTM output, two fully connected layers are stacked to further extract advanced features. The output from these layers is fed into a linear regression layer to predict the final tool wear depth, facilitating risk alerts or tool replacement notifications. The model achieves an RMSE of less than 8.1% across different datasets [138].

In robotic hand applications, size constraints often limit the manipulator. AI algorithms, particularly CNNs, are employed to predict and delineate the shapes of objects larger than the hand by identifying their contours and edges. This involves tactile sensors performing contact experiments to slide over and map the object’s surface, gathering tactile data. Deep CNNs then analyze these data, focusing on shear forces from tactile movement, to accurately predict the position and angle of the object’s contours and edges, achieving position accuracy within 3 mm and angle accuracy within 9 degrees [139].

In summary, integrating artificial intelligence with sensors for prediction enhances the accuracy and real-time capabilities of forecasts, even in complex, strongly nonlinear scenarios. However, these predictions are based on monitoring data rather than mechanistic analysis models. Therefore, the accuracy and sensitivity of model predictions for unseen data or scenarios are not guaranteed, posing significant demands on the generalization abilities and robustness of ML/DL models.

## 6. Summary and Outlook

With the advancement and proliferation of sensing technology, sensors can now collect vast amounts of data, providing rich training and application scenarios for ML/DL. Furthermore, the miniaturization of sensors, cost reduction, and the development of network connectivity technologies have led to the widespread application of sensor networks across various domains. This, driven by the need for efficient and accurate data analysis and decision-making, has further propelled the development and application of ML/DL technologies in diverse sensing scenarios.

This paper provides a comprehensive overview of the enhancements and engineering applications of sensing technologies assisted by ML/DL algorithms. These advanced algorithms, capable of autonomously analyzing large datasets and identifying complex patterns and correlations, guide the design and calibration of devices and aid in their use for compensation, identification, classification, and prediction. This significantly improves the sensors’ accuracy and adaptability to environmental changes, as detailed in Table 1.

Despite significant progress in ML/DL-guided sensing technology, several challenges remain, presenting opportunities for future research. First, the issue of data quality and quantity is paramount; high-quality, large-scale datasets with accurate annotations are fundamental to the successful application of ML and DL in sensing technology. However, data acquisition often relies on limited laboratory testing of sensors, which may lead to overfitting of the algorithms. Additionally, in the device design and calibration process, reliance on numerical simulations means the accuracy and comprehensiveness of numerical models significantly impact device performance. Second, the generalization ability of models—ensuring models perform well on unseen data—is an ongoing challenge. Error compensation, recognition, and prediction driven by AI in sensor applications are based on test data, meaning the impact of performance variations throughout the sensor’s lifecycle on these outcomes cannot be fully captured in tests, placing high demands on model generalization. Moreover, device power consumption is a concern; running complex DL models on sensor chips for compensation, recognition, or prediction while ensuring accuracy and real-time response, especially in multi-sensor decision-making and long-term monitoring, challenges sensor power efficiency. Lastly, model interpretability—the understanding and explanation of a model’s decision-making process—is crucial for the iterative optimization of sensors and their broader application across more scenarios. To address these issues, it is necessary to employ techniques such as data augmentation [140] and adversarial training [141] during the data collection process to optimize the quality and quantity of datasets. Additionally, introducing noise and interference during model training can enhance the generalization capability to unknown data. Furthermore, the development of multi-field coupled simulation methods is essential to improve the comprehensiveness and accuracy of numerical data while enhancing the interpretability of DL/ML models. Finally, advancements in model compression [142] and edge computing [143] technologies are needed to reduce model complexity and offload certain model computation tasks to the device side, thereby reducing device power consumption.

In addition to addressing the challenges related to algorithms, sensors, and their integration, several other research directions are crucial for advancing AI-driven sensing technology. One significant segment of research interest is the development of physics-based ML/DL models, such as Physics-informed Neural Network (PINN) [144], which enhance model accuracy, reduce sample size, and improve decision-making transparency by applying constraint equations to the processes of error compensation, classification, and prediction in sensors, considering the multi-field coupling within sensors and their interaction with the environment. Furthermore, developing more biocompatible materials for sensor data processing could enable near-sensor and in-sensor computing [145,146] for implantable devices, presenting a significant advance in AI-driven sensing technology.

## Figures and Tables

**Table 1:** Summary of the impact of ML/DL on sensing technology.

		Sensor Category	AI Methods	Accuracy (%)	Advantage	Disadvantage	Reference
**Sensor design**		Pressure sensor	MLP	99	1. Reduce design time and costs; 2. Enhance sensitivity; 3. Improve signal-to-noise ratio and increase precision.	1. Require substantial training data; 2. Unable to predict performance changes over time.	[44,45,48,51,52,54]
			KNN, LDA, DT	99			
			FLANN, BP	97			
		Fiber Bragg grating (FBG) sensor	GBR	90			
			RFR, GBR, ABR	90			
**Calibration and compensation**		Capacitive pressure sensor	MLP	99.5	1. Enhance calibration accuracy and speed while reducing calibration costs; 2. Minimize sensor drift during operation.	1. Require substantial training data; 2. Lacks interpretability for guiding sensor design improvements; 3. Potentially underperform in new environments.	[40,56,57,60,63,66]
			FLANN	98			
			RSNN	75			
		Peizoresistive pressure sensor	ANN	98			
			ANN	99.9			
		Fiber ring-down pressure sensor	ANN	95			
		Inertial sensor	CNN	80			
		Temperature sensor	MLP, RBF, BP	99.83			
**Object recognition and classification**	**Unidimensional data**	Pressure sensor	RF	98.93	1. Increase classification accuracy; 2. Reduce recognition errors due to environmental changes.	1. Insufficient training data can lead to overfitting; 2. Challenging to identify the optimal recognition model structure.	[69,72,80,94,105,112]
		Flexible full-textile pressure sensor	CNN	93.61			
		Textile triboelectric sensor	SFNN	98.8			
		Bioelectric sensor	SVM	92			
		Inertial sensor	MPNN	95			
		Acoustic sensor	GMM	97.5			
		Vibration sensor	GDBM	95.17			
		Vibrotactile sensor	KNN	97			
	**Multi-dimensional data**	Pressure sensor + acceleration sensor	CNN	94.4	1. Enhance classification accuracy; 2. Handle multi-source data for complex recognition tasks; 3. Ensure rapid response for real-time processing.	Sensor placement significantly impacts recognition outcomes.	[119,122,123,126,130,132]
			RF, SVM, LightGBM	90.9			
		Pressure sensor + material sensor	MLP	98.9			
		Pressure sensor + strain sensor	LSTM	97.13			
		Strain sensor + position sensor	DNN	99			
		Strain sensor + composite piezoresistive sensor	SVM	92			
		Temperature sensor + deformation sensor	CNN	86.3			
		Carbon dioxide sensor + temperature-humidity sensor + metal oxide semiconductor sensor	SVM-RFE, CBR	95			
**Prediction**		Pressure sensor	MR, TREE, FRST	94.1	1. Improve prediction accuracy and real-time capabilities; 2. Address complex nonlinear forecasting issues.	Limited generalizability and robustness, with unknown predictive capability for untrained scenarios.	[18,133,135,137,138,139]
			KNN	93			
			SVM, RF, LR, NB	81			
			CNN, AE	92			
		Iontronic pressure sensor	FCN	91.8			
		Gas sensor	CNN-LSTM	93.9			

## Data Availability

Data sharing is not applicable.
